# A hierarchical combination algorithm for real-time cycle slip detection and repair in low satellite elevation and high ionospheric activity conditions

**DOI:** 10.1038/s41598-024-52902-x

**Published:** 2024-01-29

**Authors:** Haofei Ban, Kezhao Li, Kai Wang, Yingxiang Jiao, Lingfeng Liang, Chendong Tian, Zhe Yue

**Affiliations:** 1 School of Surveying and Land Information Engineering, Henan Polytechnic University, Jiaozuo, 454000 China; 2 Collaborative Innovation Center of BDS Research Application, Zhengzhou, 450052 China

**Keywords:** Aerospace engineering, Civil engineering

## Abstract

To enhance the accuracy and robustness of cycle slip detection and repair for triple-frequency data while minimizing the adverse effects of low satellite elevation and high ionospheric activity, a hierarchical combination algorithm for real-time cycle slip detection and repair is proposed. This algorithm begins by prioritizing the reduction of noise and ionospheric delay coefficients. It determines the optimal coefficients for the combination of observations from the BeiDou Navigation Satellite System’s (BDS) Extra-Wide Lane (EWL), Wide Lane (WL), and Narrow Lane (NL). Leveraging the longer wavelength characteristics of the EWL combination, it simultaneously conducts cycle slip detection on the EWL combination alongside the pseudorange combination. Following this, based on the detection outcomes from the EWL combination, cycle slip detection is carried out on the WL combination. Finally, using the detection findings from the WL combination, cycle slip detection is executed on the NL combination. Given the NL combination’s shorter wavelength and higher susceptibility to ionospheric delay, a dynamic ionospheric prediction model is applied to the NL combination to further mitigate the impact of ionospheric disturbances. After completing the cycle slip detection process, the results from the EWL, WL, and NL combinations are integrated and solved. Experimental results clearly demonstrate that, even in scenarios characterized by low satellite elevation and active ionospheric conditions, this algorithm consistently delivers outstanding detection performance for cycle slip instances, particularly for small cycle slip (less then two cycles). Remarkably, this performance is achieved without the need for intricate searches during cycle slip repair.

## Introduction

The BeiDou Navigation Satellite System (BDS) has injected fresh vigor into the advancement of Global Navigation Satellite Systems (GNSS). BDS boasts widespread applications in diverse domains such as aviation, maritime navigation, vehicle navigation, surveying, and geographic information systems^1–4^. Nevertheless, the practical use of GNSS receivers is often fraught with challenges due to the multitude of error sources during signal propagation. One such significant challenge is the cycle slip issue. A cycle slip denotes a sudden and discontinuous shift in the phase of satellite signals received by a GNSS receiver, stemming from various causes^5,6^. This phase disruption can result in inaccuracies in positioning, subsequently compromising navigation precision and dependability. To confront the cycle slip predicament, numerous researchers have put forth various cycle slip detection and repair methodologies. Common cycle slip detection approaches include those grounded in combination models, statistical features, and filtering techniques.

The cycle slip detection method based on combination models identifies cycle slips by skillfully amalgamating diverse models or multiple data sources to establish a comprehensive observation equation or parameter inversion model. This encompasses techniques such as the TurboEdit algorithm^7–9^, the pseudorange phase combination method^10–12^, and the ionospheric residual method^13–15^, among others. The TurboEdit algorithm merges the Melbourne-Wübbena (MW) combination with the Geometry-Free (GF) combination, effectively mitigating the impact of insensitive cycle slips and delivering robust detection accuracy. Nevertheless, due to substantial pseudorange noise, the TurboEdit algorithm may struggle to detect small cycle slips lasting only 1–2 cycles; The pseudorange phase combination method is less susceptible to various errors and enjoys widespread adoption. However, due to the influence of ionospheric delay and pseudorange noise, it may not accurately detect small cycle slips, especially in situations with low elevation; The Phase Ionospheric Residual (PIR) method employs ionospheric combinations for cycle slip detection and repair, making it well-suited for identifying small cycle slips. However, in the presence of high ionospheric activity, the accuracy of cycle slip detection using this method may be compromised. Wang et al^16^. combined the GIGF combination and the quadratic difference between epochs to further improve the applicability of the TurboEdit algorithm during ionospheric active periods. However, the method is greatly affected by observation noise, and due to its uneven combination coefficients being integers, it requires the assistance of search algorithms in the cycle slip repair process. Huang et al^17^. proposed an instantaneous triple-frequency cycle slip detection and repair method that applies two geometry-free phase combinations and one geometry-free pseudorange minus phase linear combination for detecting insensitive cycle slips and uses the least-squares ambiguity decorrelation adjustment (LAMBDA) algorithm to search for cycle slip candidates. However, this method is susceptible to the influence of high ionospheric activity.

Statistical feature algorithms harness the statistical characteristics of signals, such as signal variance or higher-order moments, to pinpoint cycle slip events. These include techniques like wavelet transforms^18^ and polynomial fitting methods^19–21^, etc. The wavelet transform method is sensitive to small cycle slips but may exhibit reduced efficiency in cycle slip correction, making it less suitable for rapid processing; Huo et al^22^. studied the characteristics of non differential observation data and introduced SA4 wavelet to detect cycle slips, further improving the detection ability for small cycle slips. The polynomial fitting method detects cycle slips by fitting and calculating carrier phase sequence values, assessing whether the anomaly coincidence point signifies a cycle slip based on the consistency between the fitted curve and the measured curve. This method demands relatively high data sampling accuracy. Zhang et al^23^. used multiple time periods in the time difference model for cycle slip estimation and achieved good cycle slip repair results.

Filtering techniques employ filters to smooth signals and detect cycle slips by analyzing the residuals post-filtering. These encompass high-order differencing^24,25^ and Kalman filtering^26,27^, etc. High-order differencing stands out for its simplicity and absence of complex matrix operations, rendering it straightforward to implement. However, its detection process involves basic differencing and filtering, making it less suitable for identifying small cycle slips. Cai et al^28^. proposed an improved method that combined ionospheric residual and high-order difference. By combining the high-pass filtering characteristics of high-order differences and ionospheric residuals, the first-order difference is calculated to effectively suppress low-frequency signals and eliminate constant parts, amplifying the actual impact of cycle slips and improving the accuracy of cycle slip detection; Kalman filtering is an optimal filtering algorithm used to estimate the state of dynamic systems. While it can achieve high-precision cycle slip detection, it necessitates a high level of accuracy in system modeling and observational data quality. Liu et al^29^. employed the first-order Gauss–Markov stochastic process and Kalman filtering for real-time estimation. By conducting statistical hypothesis testing on predicted residual sequences, they successfully identified cycle slips, thereby enhancing the capability to detect such slips in various environmental conditions.

As GNSS technology continues its evolution, the introduction of new signal propagation environments and receiver technologies can pose fresh challenges concerning cycle slip occurrences. Hence, it becomes imperative to develop and fine-tune cycle slip detection and repair algorithms specifically tailored to these novel scenarios. How can it be more suitable for the use of the three types of BDS satellites in environments with low satellite elevation and high ionospheric activity? Alternatively, complex search steps can be omitted during cycle slip repair. In response to this need, a hierarchical combination for real-time cycle slip detection and repair has been introduced, with the primary objective of enhancing the precision and resilience of cycle slip handling in low satellite elevation and high ionospheric activity. Through comprehensive experimental validation and performance analysis, the efficacy of the proposed algorithm has been thoroughly examined and compared with existing methods. Research findings clearly demonstrate that the proposed algorithm maintains a high level of detection accuracy and correction precision, even in scenarios characterized by low satellite elevation and high ionospheric activity. This underscores its considerable potential for practical applications in challenging GNSS environments.

## Theory and methods

### BDS triple-frequency basic combination

The pseudorange and carrier observation equations for BDS are expressed as follows:

1 $$ P_{n} = \rho + c(\delta T_{r} - \delta T) + \gamma_{{P_{n} }} \delta I_{1}^{{}} + trop + \varepsilon_{{P_{n} }} $$

2 $$ l_{n} = \lambda_{n} \varphi_{n}^{{}} = \rho + c(\delta T_{r} - \delta T) - \gamma_{{\varphi_{n} }} \delta I_{1}^{{}} + trop - \lambda_{n} N_{n}^{{}} (t_{0} ) + \varepsilon_{{\varphi_{n} }} $$

where:$n = 1,2,3$ denotes the different frequencies of BDS. For IGSO/MEO satellites, they represent the L1X, L5X, and L6I respectively, and for MEO satellites, they represent the L2I, L7D, and L6I respectively;$\rho$ denotes the geometric distance between the receiver antenna and the satellite;$c$ denotes the speed of light;$\delta T_{r}$ and $\delta T$ denote the receiver clock and the satellite clock, respectively;$P$ and $\varphi$ denote pseudorange observations and carrier phase observations, respectively;$\gamma$ denotes the ionospheric delay coefficient;$\delta I$ and $trop$ denote the ionospheric and the tropospheric delays, respectively; $\lambda$ denotes carrier wavelength; $N(t_{0} )$ denotes ambiguity of whole cycles ;$\varepsilon$ denotes observation noise. Combining three frequencies, the triple-frequency pseudorange and carrier combination can be represented respectively as:

3 $$ P_{(a,b,c)} = aP_{1} + bP_{2} + cP_{3} $$

4 $$ l_{(i,j,k)} = [i \cdot f_{1} \cdot \varphi_{1}^{{}} + j \cdot f_{2} \cdot \varphi_{2}^{{}} + k \cdot f_{3} \cdot \varphi_{3}^{{}} ]/f_{(i,j,k)} $$

where: $f$ denotes the carrier frequency; $(a,b,c)$ denote coefficients of the pseudorange combination; $(i,j,k)$ denote coefficients of the carrier combination; $f_{(i,j,k)} = if_{1} + jf_{2} + kf_{3}$ denotes the frequency of the carrier combination.

To reduce errors, such as the receiver and satellite clock biases, differences are computed using observations between adjacent epochs^17^. The differences in pseudorange observations between adjacent epochs of Eq. (3) are expressed as:

5 $$ \begin{gathered} \Delta P_{(a,b,c)} = a\Delta P_{1} + b\Delta P_{2} + c\Delta P_{3} \hfill \\ \, = (a + b + c)(\Delta \rho + \gamma_{P(a,b,c)} \cdot \Delta \delta I_{{}}^{S} + \sigma_{P(a,b,c)} ) \hfill \\ \end{gathered} $$

where: $\Delta$ denotes difference between adjacent epochs; $\gamma_{P}$ denotes the ionospheric delay coefficients of pseudorange combination; $\sigma_{P}$ denotes the noise of pseudorange combination.

The differences in carrier observations between adjacent epochs of Eq. (4) are expressed as

6 $$ \begin{gathered} \Delta l_{(i,j,k)} = [i \cdot f_{1} \cdot \Delta \varphi_{1}^{{}} + j \cdot f_{2} \cdot \Delta \varphi_{2}^{{}} + k \cdot f_{3} \cdot \Delta \varphi_{3}^{{}} ]/f_{(i,j,k)} \hfill \\ \, = \Delta \rho_{{}}^{{}} (t) - \gamma_{l(i,j,k)} \cdot \Delta \delta I_{1}^{{}} - \lambda_{(i,j,k)} \cdot \Delta N_{(i,j,k)}^{{}} (t0) + \sigma_{l(i,j,k)} \hfill \\ \end{gathered} $$

where: $\gamma_{l}$ denotes the ionospheric delay coefficients of carrier combination; $\lambda_{(i,j,k)}$ denotes the wavelength of the carrier combination; $N_{(i,j,k)} (t_{0} )$ denotes the ambiguity of carrier combination. $\sigma_{l}$ denotes the noise of carrier combination.

The specific expressions for each of these quantities are as shown in Table 1:

**Table 1:** Symbolic meaning.

$\lambda_{(i,j,k)} = \frac{c}{{f_{(i,j,k)} }}$	$\gamma_{P(a,b,c)} = a + b \cdot \frac{{f_{1}^{2} }}{{f_{2}^{2} }} + c \cdot \frac{{f_{1}^{2} }}{{f_{3}^{2} }}$
$\gamma_{l(i,j,k)} = \frac{{f_{1} }}{{f_{(i,j,k)} }}(i + j\frac{{f_{1} }}{{f_{2} }} + k\frac{{f_{1} }}{{f_{3} }})$	$\sigma_{P(a,b,c)}^{2} = a^{2} \varepsilon_{{_{{P_{1} }} }}^{2} + b^{2} \varepsilon_{{_{{P_{2} }} }}^{2} + c^{2} \varepsilon_{{_{{P_{3} }} }}^{2}$
$\sigma_{{_{l(i,j,k)} }}^{2} = \frac{{i^{2} f_{1}^{2} \varepsilon_{{\varphi_{1} }}^{2} + j^{2} f_{2}^{2} \varepsilon_{{\varphi_{2} }}^{2} + k^{2} f_{3}^{2} \varepsilon_{{\varphi_{3} }}^{2} }}{{f_{(i,j,k)}^{2} }}$	$\Delta N_{(i,j,k)} (t0) = iN_{1} (t0) + kN_{2} (t0) + jN_{3} (t0)$

In order to find the optimal linear combination of carriers, it is essential to establish effective selection criteria. As per Eq. (6), the residual ionospheric delay and observation noise play a crucial role in determining phase ambiguities. Taking into consideration the different combination wavelengths, the following two criteria are adopted: (1)Minimize the ionospheric delay coefficients to reduce the impact of ionospheric variability; (2)Minimize the interference of observation noise on data as much as possible. These criteria can be expressed as follows:

7 $$ \left\{ {\begin{array}{*{20}c} {(i,j,k) = \mathop {\arg }\limits_{(i,j,k)} \min \left( {\left| {\frac{{\gamma_{l(i,j,k)} }}{{\lambda_{(i,j,k)} }}} \right|} \right)} \\ {(i,j,k) = \mathop {\arg }\limits_{(i,j,k)} \min \left( {\left| {\frac{{\sigma_{{_{l(i,j,k)} }}^{{}} }}{{\lambda_{(i,j,k)}^{{}} }}} \right|} \right)} \\ \end{array} } \right. $$

Based on the geometric analysis in reference^30^, simultaneously mitigating errors caused by observation noise and ionospheric delay presents a set of conflicting factors. Therefore, in practice, there is a need to strike a balance between these two factors by selecting suitable combination coefficients through a compromise. To limit the error magnitude, integer coefficients should be determined within the range of − 5 to 5. Assuming that the carrier observation noise for the three frequencies is independently and identically distributed with the same standard deviation^31,32^, and given that the carrier phase noise for different BDS frequency bands is the same(i.e.,$\varepsilon_{{\varphi_{1} }} = \varepsilon_{{\varphi_{2} }} = \varepsilon_{{\varphi_{3} }} = 0.002m$),the coefficients $l_{(0, - 1,1)}^{{}}$, ,$l_{(1,0, - 1)}^{{}}$ and $l_{(1,0,0)}^{{}}$ are selected as the best combination based on the selection criteria. The BDS carrier frequencies are L1X, L2I, L5X, L6I, and L7D, with IGSO/MEO satellites using L1X, L5X, and L6I frequencies, and GEO satellites using L2I, L7D, and L6I frequencies. The Extra-Wide Lane (EWL), Wide Lane (WL), and Narrow Lane (NL) combinations parameters for the three-frequency BDS signals are as shown in Table 2.

**Table 2:** Linear combination of triple frequency BDS signals.

Satellite type	Combination type ($i,j,k$)	$\gamma_{l}$	$\sigma_{{_{l} }}^{{}}$	$\lambda_{(i,j,k)} (m)$
GEO (IGSO/MEO)	EWL (0, − 1,1)	− 1.591 (− 1.663)	0.057 (0.037)	4.884 (3.256)
	WL (1,0, − 1)	− 1.293 (− 1.241)	0.011 (0.013)	0.846 (0.976)
	NL (1,0,0)	1.000 (1.000)	0.002 (0.002)	0.192 (0.190)

According to Eq. (5), pseudorange combination is primarily affected by pseudorange observation noise. Similar to carrier observation noise^33^, assume that the pseudorange noise for BDS is the same (i.e.,$\varepsilon_{{P_{1} }} = \varepsilon_{{P_{2} }} = \varepsilon_{{P_{3} }} = \varepsilon_{{P_{{}} }} = 0.3m$). Referring to Table 1, it is apparent that when $a = b = c = 1/3$, the combination noise is minimized. Therefore, selecting $P_{(1/3,1/3,1/3)}$ as the pseudorange combination coefficient. After determining the coefficients for carrier and pseudorange combination, a hierarchical combination model is established to facilitate cycle slip detection and repair.

### The hierarchical combination model

#### EWL combination cycle slip detection model

The EWL combination retains the integer cycle slips of the combination ambiguity, and since the wavelength is large, it is not affected much by the residual errors. First, cycle slips of the EWL combination are detected and repaired. Combined with the pseudorange combination, the pseudorange and carrier phase combination is constructed according to Eqs. (5) and (6), which is expressed as:

8 $$ \begin{gathered} \Delta N_{(0, - 1,1)} = \Delta l_{(0, - 1,1)}^{{}} - \Delta P_{(1/3,1/3,1/3)}^{{}} \hfill \\ \, = \left[ {(f_{2} \Delta \varphi_{2}^{{}} - f_{3} \Delta \varphi_{3}^{{}} )/(f_{2} - f_{3} ) - (\Delta P_{1}^{{}} + \Delta P_{2}^{{}} + \Delta P_{3}^{{}} )/3} \right]/\lambda_{(0, - 1,1)} + \eta_{1} \Delta \delta I_{1} + \sigma_{1} \hfill \\ \end{gathered} $$

9 $$ \left\{ {\begin{array}{*{20}c} {\eta_{1} = - (\gamma_{(0, - 1,1)} + \gamma_{(1/3,1/3,1/3)} )/\lambda_{(0, - 1,1)} } \\ {\sigma_{1} = \sqrt {\sigma_{{P_{(1/3,1/3,1/3)} }}^{2} + \sigma_{{l_{(0, - 1,1)} }}^{2} } /\lambda_{(0, - 1,1)} } \\ \end{array} } \right. $$

The ionospheric coefficients $\eta_{1}$ for GEO satellites and IGSO/MEO satellites in the EWL combination are 0.040 and 0.066, respectively. Correspondingly, the observation noise values $\sigma_{1}$ are 0.037 and 0.054, respectively. When the EWL combination observation exceeds 0.5, indicating that $\left| {\Delta N_{(0, - 1,1)} } \right| > 0.5$, it is considered a cycle slip. In such instances, the cycle slip value for the EWL combination is simply rounded to the nearest integer, denoted as $\Delta \hat{N}_{(0, - 1,1)} = round[\Delta N_{(0, - 1,1)} ]$. Given the negligible magnitude of $\eta_{1}$, it can effectively be disregarded when using a 30 s sampling interval^31^. Consequently, the success rate of cycle slip detection for the EWL combination is as follows:

10 $$ \begin{gathered} P_{1} = P\left( {\left| {\Delta N_{(0, - 1,1)} - \Delta \hat{N}_{(0, - 1,1)} } \right| < 0.5} \right) \hfill \\ { = }\Phi \left( {\frac{{0.5 - \eta_{1} \cdot \Delta \delta I_{1} }}{{\sigma_{1} }}} \right) + \Phi \left( {\frac{{0.5 + \eta_{1} \cdot \Delta \delta I_{1} }}{{\sigma_{1} }}} \right) - 1 = 100\% \hfill \\ \end{gathered} $$

#### WL combination cycle slip detection model

After the cycle slip of the EWL combination is determined, the repaired EWL combination is utilized to detect the observations of the WL combination:

11 $$ \Delta N_{(1,0, - 1)} = \frac{1}{{\lambda_{(1,0, - 1)} }}(\Delta l_{(1,0, - 1)}^{{}} - \Delta l_{(0, - 1,1)}^{{}} + \lambda_{(0, - 1,1)} \Delta \hat{N}_{(0, - 1,1)} ) + \eta_{2} \Delta \delta I_{1}^{S} + \sigma_{2} $$

12 $$ \left\{ {\begin{array}{*{20}c} {\eta_{2} = ( - \gamma_{(1,0, - 1)} + \gamma_{(0, - 1,1)} )/\lambda_{(1,0, - 1)} } \\ {\sigma_{2} = \sqrt {\sigma_{{l_{(1,0, - 1)} }}^{2} + \sigma_{{l_{(0, - 1,1)} }}^{2} } /\lambda_{(1,0, - 1)}^{{}} } \\ \end{array} } \right. $$

The ionospheric coefficients $\eta_{2}$ for GEO satellites and IGSO/MEO satellites in the WL combination are -0.352 and -0.431, respectively. Correspondingly, the observation noise values $\sigma_{2}$ are 0.068 and 0.040 respectively. When the WL combination observation exceeds 0.5, indicating that $\left| {\Delta N_{(1,0, - 1)} } \right| > 0.5$, it is considered a cycle slip. In such instances, the cycle slip value for the WL combination is simply rounded to the nearest integer, denoted as $\Delta \hat{N}_{(1,0, - 1)} = round[\Delta N_{(1,0, - 1)} ]$.Similarly to the EWL combination, since the $\eta_{2}$ value remains small, it can be ignored with a 30 s sampling interval for WL combination. Therefore, the success rate of cycle slip detection for the WL combination is:

13 $$ \begin{gathered} P_{2} = P\left( {\left| {\Delta N_{(1,0, - 1)} - \Delta \hat{N}_{(1,0, - 1)} } \right| < 0.5} \right) \hfill \\ { = }\Phi \left( {\frac{{0.5 - \eta_{2} \cdot \Delta \delta I_{1} }}{{\sigma_{2} }}} \right) + \Phi \left( {\frac{{0.5 + \eta_{2} \cdot \Delta \delta I_{1} }}{{\sigma_{2} }}} \right) - 1{\text{ = 100\% }} \hfill \\ \end{gathered} $$

#### NL combination cycle slip detection model

After the cycle slip of the WL combination is determined, the repaired WL combination is utilized to detect the observations of the NL:

14 $$ \Delta N_{(1,0,0)} = \frac{1}{{\lambda_{(1,0,0)} }}(\Delta l_{(1,0,0)}^{{}} - \Delta l_{(1,0, - 1)}^{{}} + \lambda_{(1,0, - 1)} \Delta \hat{N}_{(1,0, - 1)} ) + \eta_{3} \cdot \Delta \delta I_{1}^{{}} + \sigma_{3} $$

15 $$ \left\{ {\begin{array}{*{20}c} {\eta_{3} = ( - \gamma_{(1,0,0)} + \gamma_{(1,0, - 1)} )/\lambda_{(1,0,0)} } \\ {\sigma_{3} = \sqrt {\sigma_{(1,0,0)}^{2} + \sigma_{(1,0, - 1)}^{2} } /\lambda_{(1,0,0)}^{{}} } \\ \end{array} } \right. $$

The ionospheric coefficients $\eta_{3}$ for GEO satellites and IGSO/MEO satellites in the NL combination are − 11.941 and − 11.781, respectively. The observation noise values $\sigma_{3}$ are 0.058 and 0.070, respectively. It is evident that when the value of $\eta_{3}$ experiences a significant increase, direct integer rounding of NL's cycle slip values may not be suitable during periods of heightened ionospheric activity. Polynomial regression models are commonly employed to capture temporal variations. In this context, a polynomial regression function incorporating time variables can be introduced to model the time series of NL combination values. This facilitates the prediction of epoch-to-epoch ionospheric delay within the NL combination, thereby mitigating the influence of ionospheric delay. The general form of a polynomial regression fit with a window size of $n$ and a polynomial order of $p$ is as follows:

16 $$ \left\{ {\begin{array}{*{20}c} {y(t) = x_{0}^{{}} + x_{1}^{{}} \Delta t(1) + x_{2}^{{}} \Delta t(2)^{2} + \cdots + x_{p}^{{}} \Delta t(i)^{p} } \\ {\Delta t(i) = t_{i} - t_{i - 1} } \\ \end{array} } \right. $$

where: $i = 1,2,3, \cdots ,m$;$x$ and $y$ denote polynomial regression coefficients and NL combination observations, respectively. $t$ denotes the time of the epoch. The choice of the polynomial fit order $p$ in polynomial regression should be based on the variations of each NL combination value with respect to time within the data cycle. According to prior research: During periods of active ionospheric conditions, $p$ is set to 2. And during stable ionospheric conditions, $p$ is set to 1^34^. The decreasing correlation between observations over time has been confirmed through multiple tests. It has been found that a time interval of 5 to 15 min is sufficient to meet the requirements of the prediction window under normal conditions. However, when satellite elevation is relatively low, and noise effects are more pronounced, longer time intervals are needed for prediction. Therefore, in the calculation of ionospheric delay, a dynamic window of length $m$ is employed, with the maximum window size set to $m_{\max }$ = 30. The specific implementation strategy is as follows:

1. When the receiver initially receives satellite signals, especially when the satellite elevation is low, and effective data support is needed for subsequent NL combination observations detection, the window size should be as large as possible. Therefore, in this case, the window size is set to the maximum window count, which is $m = m_{\max }$;
2. When the satellite elevation $EL$ is greater than $30^\circ$, the variations in multipath error and noise error tend to be relatively smooth. Therefore, in this situation, it is advisable to use a fixed window size for data processing, which is typically expressed as $m = 0.5m_{\max }$.
3. When $EL$ is less than $30^\circ$ but greater than $15^\circ$, where noise error has a significant impact, it is advisable to dynamically adjust the window size to better accommodate the changing elevation. In this case, the window size can be set as $m = m_{\max } (1 - \sin el)$.
4. When $EL$ is less than $15^\circ$, noise error significantly increases, and in such conditions, it is advisable to use the maximum window size. Additionally, when $EL$ is less than $10^\circ$, it becomes challenging to capture satellite signals, and the signal quality is very poor. Therefore, for $EL$ less than $15^\circ$ greater than $10^\circ$ the window size can be set as $m = m_{\max }$.

In summary, the dynamic window size can be expressed as follows:

17 $$ m = \left\{ {\begin{array}{*{20}c} {m_{\max } \, i \le m_{\max } } \\ {0.5m_{\max } \, EL \ge 30^\circ ,i > m_{\max } } \\ {m_{\max } (1 - \sin el) \, 15^\circ \le EL < 30^\circ ,i > m_{\max } } \\ {m_{\max } { 10}^\circ \le EL < 15^\circ ,i > m_{\max } } \\ \end{array} } \right. $$

After determining the order of polynomial fitting and the window, the following equation can be derived based on Eq. (16):

18 $$ \left( {\begin{array}{*{20}c} 1 & {\Delta t(i - m + 1)} & \cdots & {(\Delta t(i - m + 1))^{p} } \\ 1 & {\Delta t(i - m + 2)} & \cdots & {(\Delta t(i - m + 2))^{p} } \\ \vdots & \vdots & \vdots & \vdots \\ 1 & {\Delta t(i)} & \cdots & {(\Delta t(i))^{p} } \\ \end{array} } \right)\left( {\begin{array}{*{20}c} {x_{0}^{{}} } \\ {x_{1}^{{}} } \\ \vdots \\ {x_{p}^{{}} } \\ \end{array} } \right) = \left( {\begin{array}{*{20}c} {\Delta N_{(1,0,0)} (i - m + 1)} \\ {\Delta N_{(1,0,0)} (i - m + 2)} \\ \vdots \\ {\Delta N_{(1,0,0)} (i)} \\ \end{array} } \right) $$

Equation (18) can be simplified into matrix form as ${\varvec{A}}_{NL} {\varvec{X}}_{NL} = {\varvec{Y}}_{NL}$. Subsequently, the error equation matrix can be expressed as ${\varvec{V}}_{NL} = {\varvec{AX}}_{NL} - {\varvec{Y}}_{NL}$. Employing the least squares principle and the function-free extrema algorithm, we can eliminate ${\varvec{V}}_{NL}$ to obtain the polynomial fitting coefficients $\hat{\user2{X}}_{NL}$, denoted as:

19 $$ \hat{\user2{X}}_{NL} = ({\varvec{A}}_{{_{NL} }}^{\text{T}} {\varvec{A}}_{NL} )^{ - 1} - {\varvec{A}}_{{_{NL} }}^{\text{T}} {\varvec{Y}}_{NL} $$

After obtaining the polynomial fitting coefficients, the predicted ionospheric layer delay of the ($m + 1$)th epoch can be expressed as:

20 $$ I_{NL} (i_{m + 1} ) = \hat{x}_{0}^{{}} + \hat{x}_{1}^{{}} \Delta t(i_{m + 1} ) + \hat{x}_{2}^{{}} \Delta t(i_{m + 1} )^{2} + \cdots + \hat{x}_{p}^{{}} \Delta t(i_{m + 1} )^{p} $$

At the same time, in order to ensure the accuracy of ionospheric prediction results, it should be ensured that there is no cycle slip in the data within the initial window. The judgment algorithm is as follows:

1. According to the Eq. (19), obtain the polynomial fitting coefficients $$\overline{\user2{X}}_{NL} = \left[ {\begin{array}{*{20}c} {\overline{x}_{0}^{{}} } & {\overline{x}_{1}^{{}} } & \cdots & {\overline{x}_{p}^{{}} } \\ \end{array} } \right]^{\text{T}}$$ within the initial window, then the predicted ionospheric delay $\overline{I}_{NL}$ within the initial window can be expressed as $\overline{I}_{NF} (i) = \overline{x}_{0}^{{}} + \overline{x}_{1}^{{}} \Delta t(i) + \overline{x}_{2}^{{}} \Delta t(i)^{2} + \cdots + \overline{x}_{p}^{{}} \Delta t(i)^{p}$;
2. If the NL combination observations within the initial window satisfy:$|\Delta N_{(1,0,0)} - \overline{I}_{NF} (i)| < 0.5$.

Finally, the cycle slip value of NL combination can be expressed as:

21 $$ \Delta \hat{N}_{(1,0,0)} = round[\Delta N_{(1,0,0)} - I_{NL} ] $$

#### Cycle slip repair

Solving for cycle slips $\hat{N}$ on three frequencies using the hierarchical combination model:

22 $$ \left( {\begin{array}{*{20}c} 0 & { - 1} & 1 \\ 1 & 0 & { - 1} \\ 1 & 0 & 0 \\ \end{array} } \right)\left( {\begin{array}{*{20}c} {\hat{N}_{1} } \\ {\hat{N}_{2} } \\ {\hat{N}_{3} } \\ \end{array} } \right) = \left( {\begin{array}{*{20}c} {\Delta \hat{N}_{(0, - 1,1)} } \\ {\Delta \hat{N}_{(1,0, - 1)} } \\ {\Delta \hat{N}_{(1,0,0)} } \\ \end{array} } \right) $$

Simplify Eq. (20) into matrix form ${\varvec{AX}} = {\varvec{Y}}$. Since the combination coefficients and the obtained cycle slip values are integers, the rank of ${\varvec{A}}$ is 3, multiply ${\varvec{A}}_{{}}^{ - 1}$ directly to obtain the cycle slip values, i.e.,${\varvec{X}} = {\varvec{A}}_{{}}^{ - 1} {\varvec{Y}}$.

**Figure 1:**
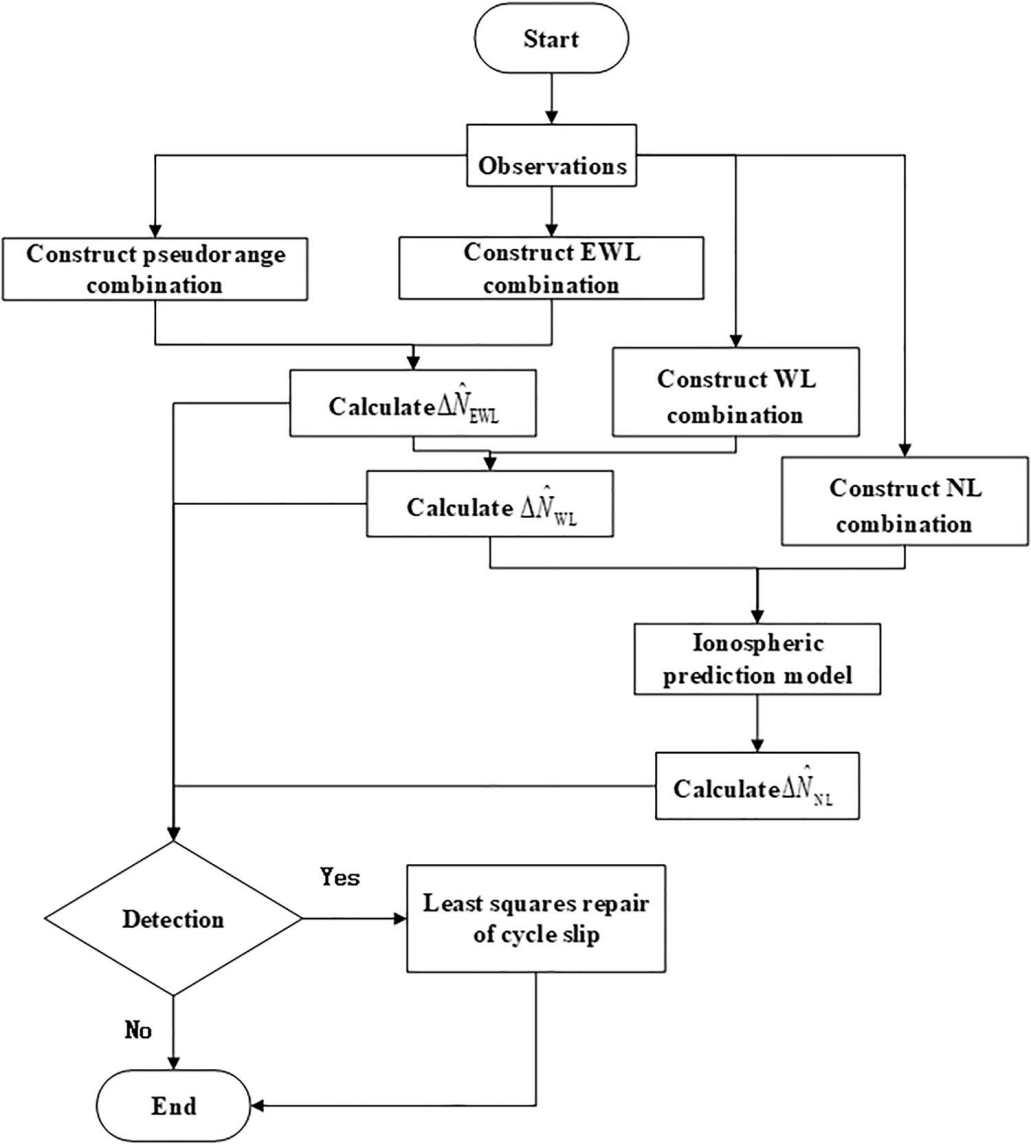
Flowchart of the cycle slip detection and repair algorithm.

The flowchart of the entire algorithm is shown in Fig. 1.

## Results and discussion

**Figure 2:**
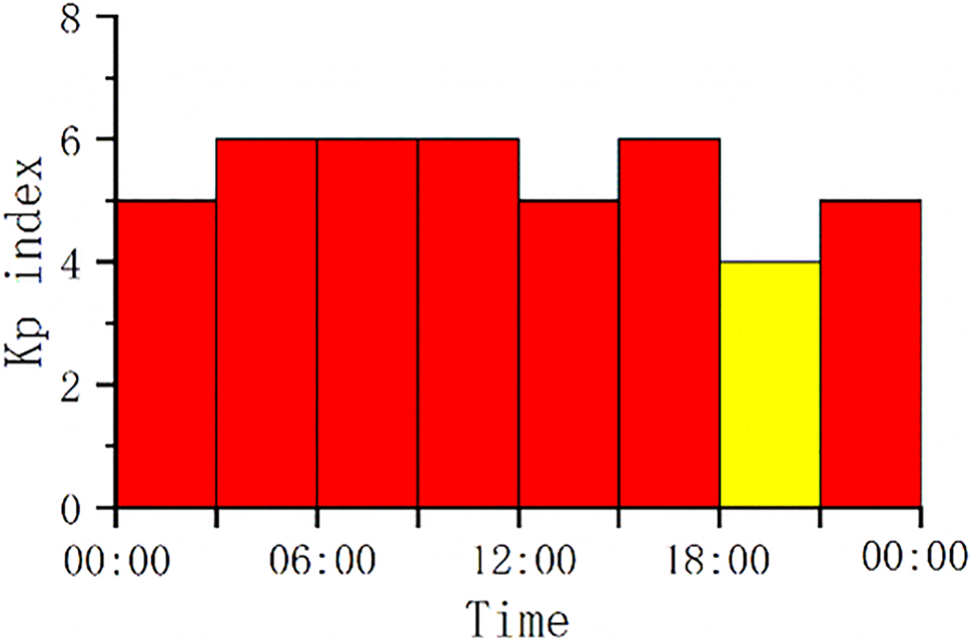
Geomagnetic Kp index.

The data comes from the IGS WUH2 station TRIMBLE ALLOY GNSS station receiver. The collection time is September 04, 2022, and the sampling rate is 30 s. A total of 2880 epochs of BeiDou satellite observation data were selected for experimental analysis. The geomagnetic Kp index on the day of the experimental data was obtained from the Space Environment Prediction Center (http://www.sepc.ac.cn) as shown in Fig. 2:

According to the calculation, the average Kp index on September 4, 2022 is 4.75, exceeding 5 most of the time, which means a major geomagnetic storm occurred that day and the ionosphere was in a violent activity state.

The experiment is divided into two schemes, as follows:

*Scheme I*: Verify with the original station data. The algorithm of hierarchical combination cycle slip detection and repair (hereinafter referred to as Algorithm 1) is directly used to detect and repair cycle slips in the original data, and compared with the three-frequency Geometry-Free and Ionosphere-Free(GFIF) combination algorithm (hereinafter referred to as Algorithm 2) detection and repair results in Reference^16^ to verify the basic performance of Algorithm 1. Algorithm 2 incorporates MW combination, GIGF combination, and PIR combination, building upon the TurboEdit algorithm, which is currently one of the most widely used cycle slip detection algorithms. In order to further ensure the accuracy of the Algorithm 2 for cycle slip repair, the least-squares ambiguity decorrelation adjustment (LAMBDA)^17^ is used to search for cycle slip candidates of the Algorithm 2. Because for a carrier data segment, the type of cycle slips occurred is singular. To further verify the applicability of Algorithm 1 to various types of cycle slips, obtain the carrier data without cycle slips through Scheme 1 for the next experiment.

*Scheme II*: Taking the carrier data without cycle slips from Scheme I as the basis, first add different types of cycle slip combinations from the ($m_{\max } + 1$)th epoch of the carrier data at intervals of 5 epochs. The first 8 cycle slip combinations are insensitive small cycle slips of EWL, WL or NL combinations, which are (1,0,0), (0,1,0), (0,0,1), (0, 2, 2), (3, 0, 3), (5, 4, 0), (5, 5, 5) and (13, 10, 0). Other cycle slip combinations are random cycle slip combinations of 0–9 cycles randomly generated for each frequency (not all zeros for the 3 frequencies). Algorithm 1 and Algorithm 2 are used to detect and repair cycle slips in the experimental data, with the purpose of fully verifying the correctness, effectiveness and applicability of Algorithm 1 in detecting and repairing different cycle slip combinations (especially insensitive cycle slips).

### Results and analysis of scheme 1

**Figure 3:**
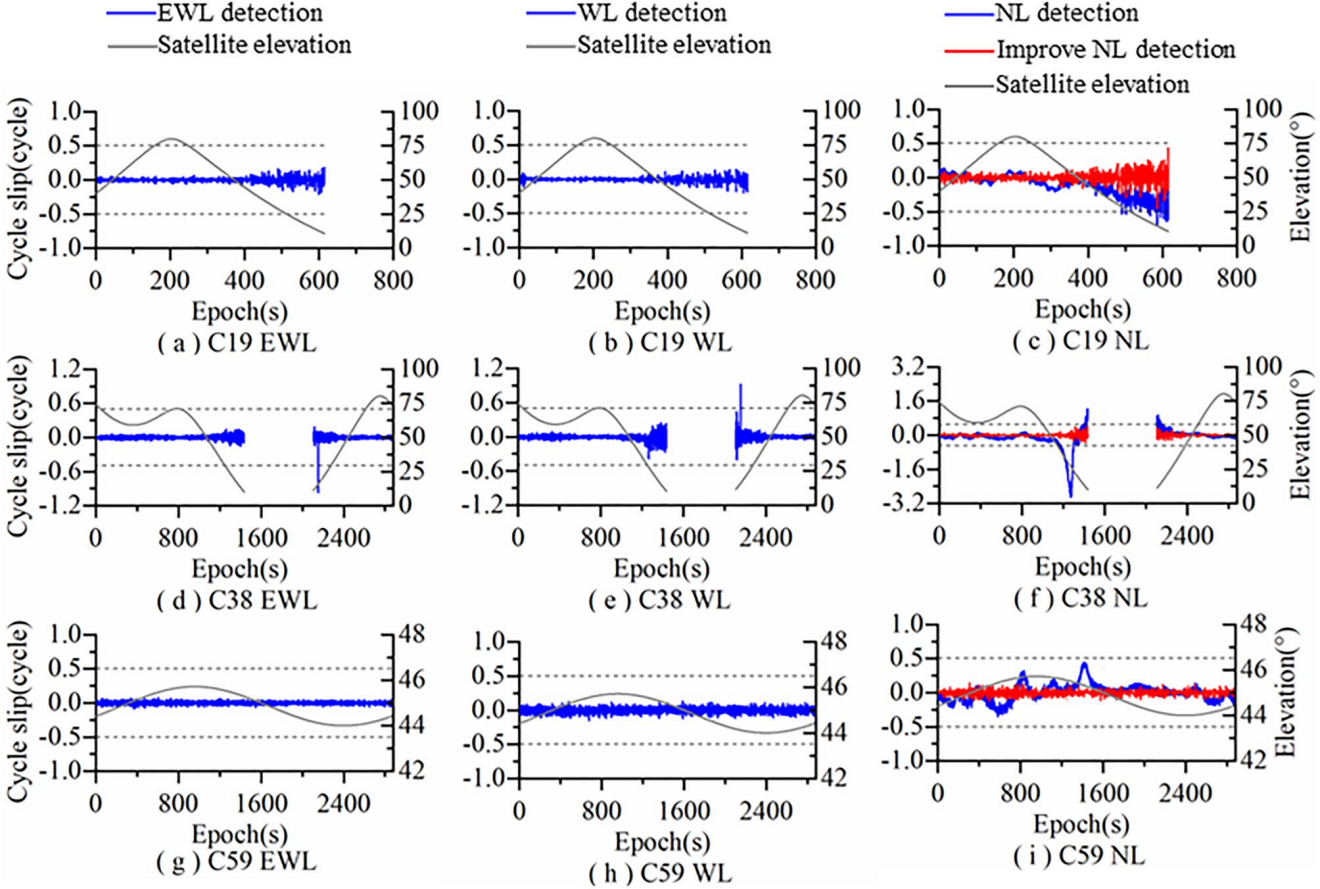
Algorithm 1 cycle slip detection results.

Figure 3 shows the detection results of Algorithm 1 for satellites C19, C38, and C59. It can be seen from Fig. 3 that the EWL combination is not affected by the satellite elevation and ionosphere, and the combination observations are within the detection threshold range without significant fluctuations. The WL combination is not affected by the ionosphere. Due to the influence of satellite elevation, the fluctuation amplitude of the combination observation increases at low elevation, but the fluctuation range is still within the detection threshold. The NL combination is affected by both elevation and ionosphere, so the fluctuation range of the combination observations changes dramatically, with a considerable portion exceeding the detection threshold. However, the NL combination improved by the dynamic ionospheric prediction model is not affected by elevation and ionosphere. Its combination observations are within the detection threshold range without significant fluctuations.

**Figure 4:**
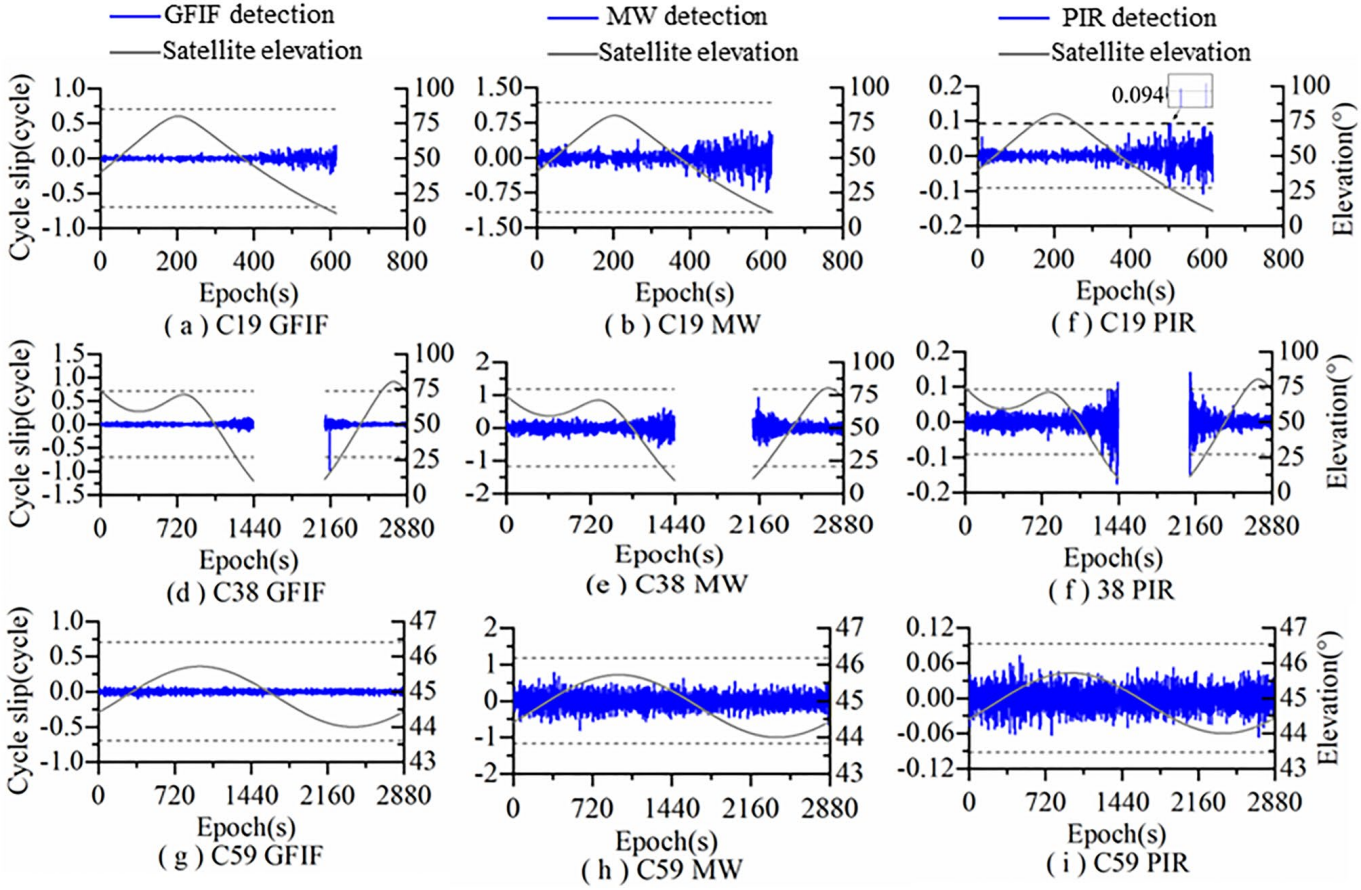
Algorithm 2 cycle slip detection results.

Figure 4 shows the detection results of Algorithm 2 for satellites C19, C38, and C59. It can be seen from Fig. 4 that the GFIF combination is not affected by satellite elevation and ionosphere. The combination observations are within the detection threshold range without significant fluctuations. The MW combination is not affected by the ionosphere. Due to the severe influence of satellite elevation, the fluctuation range of the combined values increases significantly at low elevation, with some exceeding the threshold. The PIR combination has an obviously larger fluctuation range in the combination observations due to the dual influence of elevation and ionosphere, and some of the combination observations exceed the detection threshold.

The cycle slip detection results of Algorithm 1 are shown in Table 3. Algorithm 1 did not detect cycle slips on satellites C19 and C59, and detected one group of cycle slips on satellite C38 which can be corrected successfully, without false detection. The unimproved algorithm (without using ionospheric prediction model), a total of 183 sets of cycle slips were detected, and none of them were successfully repaired. Therefore, the ionospheric prediction model can effectively weaken the impact of ionospheric activity on NL combinations, further improving the accuracy of cycle slip detection and repair.

**Table 3:** Results of using Algorithm 1 to detect and repair cycle slip for the original data.

Algorithm	Satellite	Cycle slips	Successfully repaired	Failed repaired
Algorithm 1 (Without improving)	C19- MEO	/ (12)	/ (0)	/ (12)
	C38-IGSO	1 (171)	1 (0)	0 (171)
	C59-GEO	/ (/)	/ (/)	/ (/)

The cycle slip detection results of Algorithm 2 in are shown Table 4. Algorithm 2 did not detect cycle slips on satellite C59. It detected 17 groups of cycle slips on C19 and C38, but only one set of cycle slip was successfully fixed, and the rest failed to be fixed, resulting in false detection.

**Table 4:** Results of using Algorithm 2 to detect and repair cycle slip for the original data.

Algorithm	Satellite	Cycle slips	Successfully repaired	Failed repaired
Algorithm 2 (Without LAMBDA)	C19-MEO	2 (2)	0(0)	2 (2)
	C38- IGSO	15 (15)	1 (0)	14 (15)
	C59-GEO	/ (/)	/ (/)	/ (/)

Therefore, compared with Algorithm 2, Algorithm 1 has higher accuracy and better applicability in cycle slip detection and repair. After the measured data was repaired by Algorithm 1, the changes of all combination observations were within the detection threshold. The cycle slip detection figures after repair are not shown due to limited space.

### Results and analysis of scheme II

**Figure 5:**
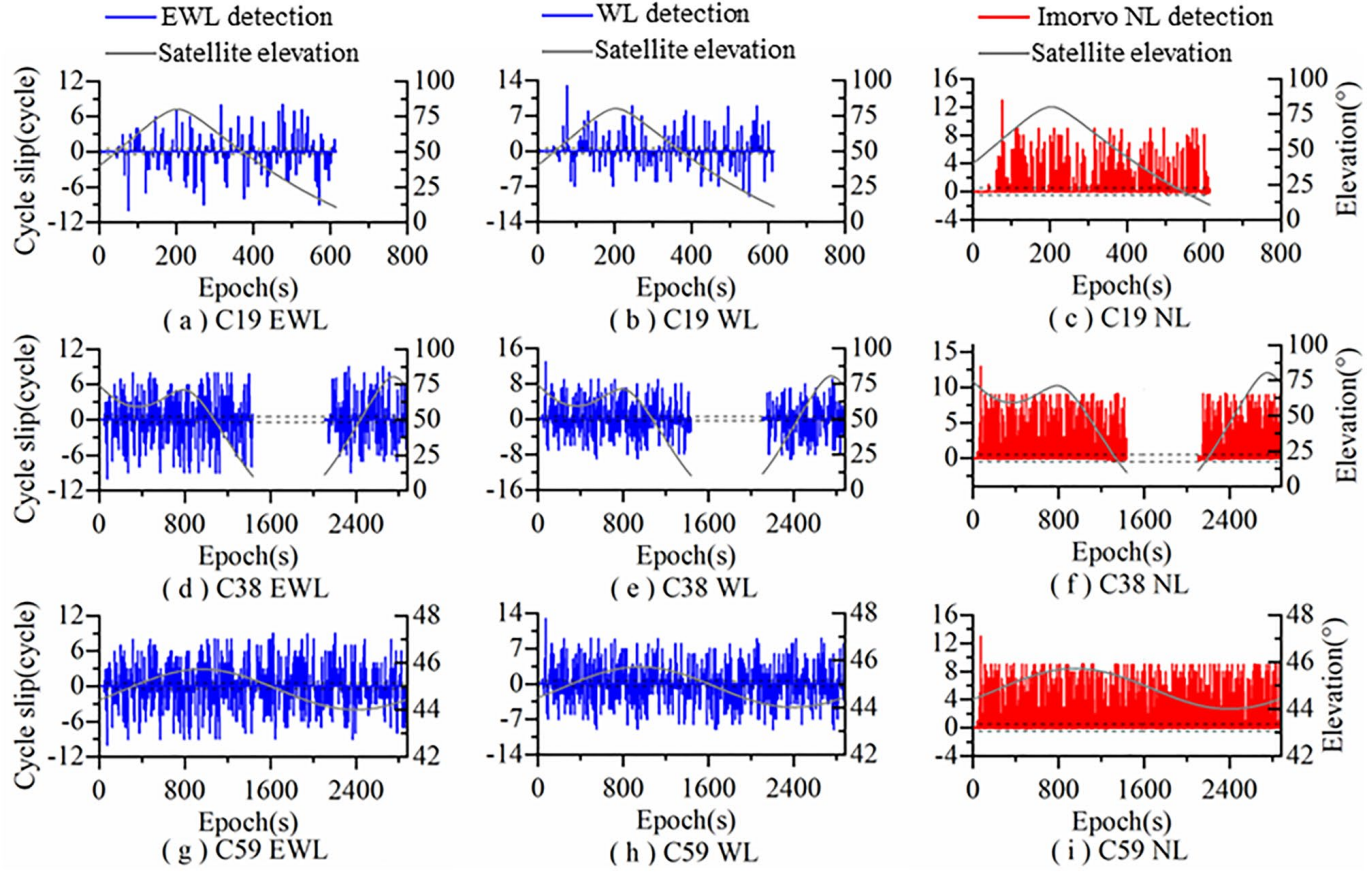
Simulated cycle slip detection results of Algorithm 1.

Figure 5 shows the detection results of Algorithm 1 for simulated cycle slips added to satellites C19, C38 and C59. It can be seen from Fig. 5 that in Algorithm 1, the EWL combination is insensitive to combined cycle slips like (1,0,0), (0,2,2) and (5,5,5), but can detect other combined cycle slips. The WL combination is insensitive to combined cycle slips like (0,1,0), (3,0,3) and (5,5,5), but can detect other combined cycle slips. The NL combination is insensitive to combined cycle slips like (0,1,0), (0,0,1), (0,2,2) and (3,0,3), but can detect other combined cycle slips. Therefore, by combining EWL, WL and NL, Algorithm 1 can detect all the added simulated cycle slips and there are no undetectable insensitive cycle slips.

**Figure 6:**
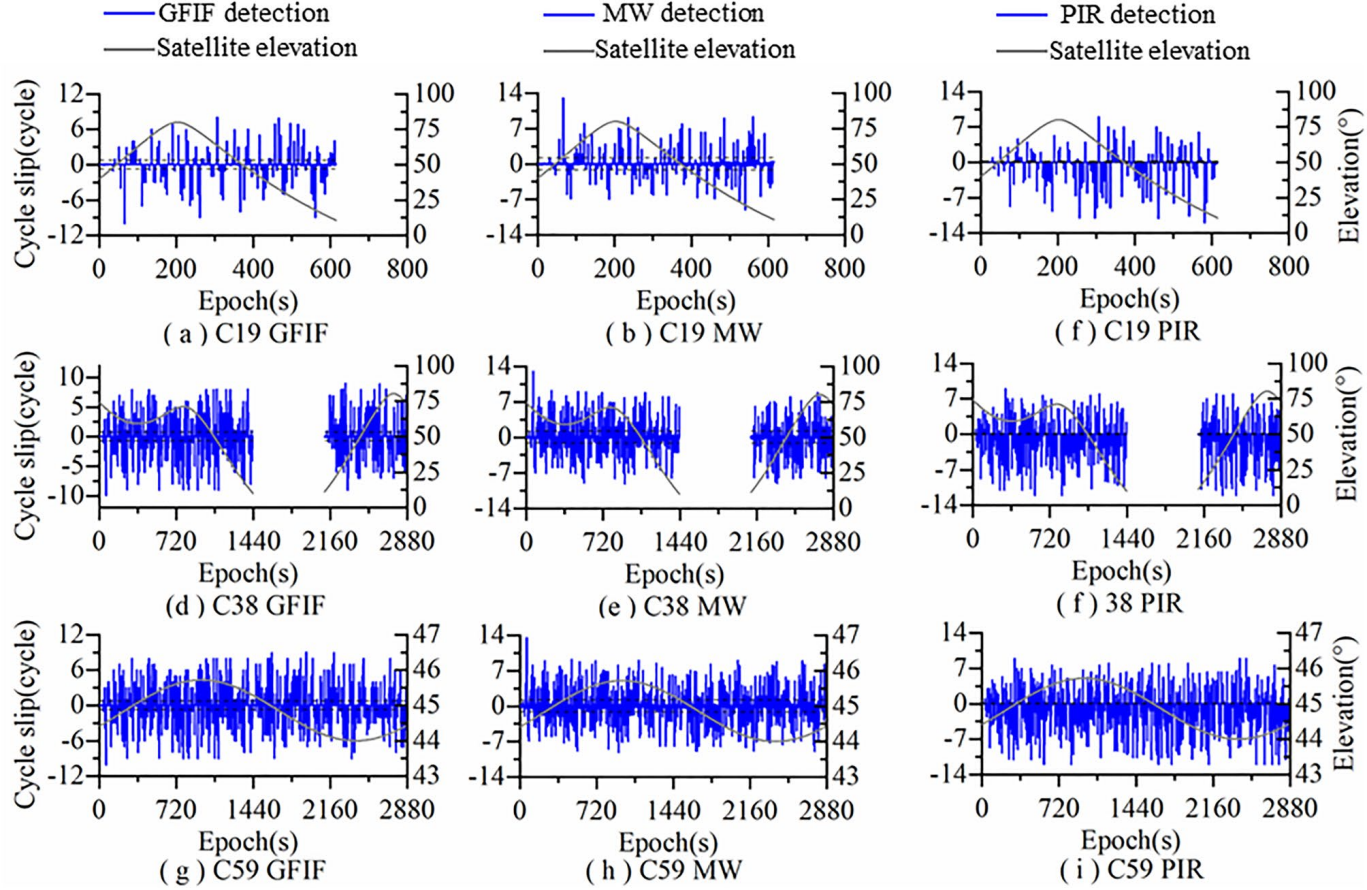
Simulated cycle slip detection results of Algorithm 2.

Figure 6 shows the detection results of Algorithm 2 for simulated cycle slips added to satellites C19, C38 and C59. It can be seen from Fig. 6 that in Algorithm 2, GFIF combination is insensitive to the combined cycle slips (1,0,0), (0,2,2) and (5,5,5), but can detect other combined cycle slips. The MW combination is insensitive to the combined cycle slips (0,1,0), (3,0,3) and (5,5,5). At the same time, due to the influence of satellite elevation, it still cannot accurately detect the remaining cycle slip combinations. The PIR combination is insensitive to the combined cycle slip (13,10,0), but still cannot detect all the remaining cycle slip combinations due to the influence of ionospheric activity. Therefore, by combining GFIF, MW and PIR, Algorithm 2 can also detect the added simulated cycle slips, but there will be false detections.

The repair results of simulated cycle slips by Algorithm 1 are shown in Table 5. Statistically, Algorithm 1 can correctly repair all the 1123 groups of simulated cycle slips added to the 3 satellites, especially for insensitive small cycle slip combinations, with a 100% correction accuracy.

**Table 5:** Simulated cycle slip repair results of Algorithm 1.

Algorithm	Satellite	Cycle slips	Successfully repaired	Failed repaired
Algorithm 1	C19-MEO	118	118	0
	C38- IGSO	434	434	0
	C59-GEO	571	571	0

The repair results of simulated cycle slips by Algorithm2 are shown in Table 6. Among the 1123 groups of simulated cycle slips added to the 3 satellites, Algorithm 2 correctly repaired 1113 groups and incorrectly repaired 10 groups, with a correction accuracy of 99.1%. Algorithm 2 without MLABDA correctly repaired 649 groups and incorrectly repaired 474 groups, with a correction accuracy of 57.79%.

**Table 6:** Simulated cycle slip repair results of Algorithm 2.

Algorithm	Satellite	Cycle slips	Successfully repaired	Failed repaired
Algorithm 2 (Without LAMBDA)	C19-MEO	118	116 (59)	2 (59)
	C38- IGSO	434	429 (263)	5 (171)
	C59-GEO	571	568 (327)	3 (244)

Comparing Algorithm 2, Algorithm 1 adopts hierarchical cycle slip detection and ionospheric prediction model to improve the accuracy of cycle slip detection while avoiding the complex search process in the traditional combination model for cycle slip repair, thus improving the correctness of cycle slip repair. Due to the fact that the combination coefficients of Algorithm 1 are all integers, there is no need for complex searches to ensure the accuracy of cycle slip repair.

## Conclusions

In this paper, an optimized algorithm for real-time hierarchical combination cycle slip detection and repair is proposed, which is not restricted by active ionospheric conditions. By using EWL, WL and NL combination models, cycle slips can be reliably detected and repaired under good conditions. However, successfully detecting and repairing cycle slips is challenging under low elevation and/or active ionospheric conditions. The newly proposed algorithm improves the cycle slip detection performance by incorporating the predicted epoch-differenced ionospheric delays to construct an ionospheric prediction model, in order to increase the accuracy of estimating cycle slip integer values.

The algorithm has many advantages. Firstly, it can detect all small cycle slips including insensitive ones. Secondly, even with rapidly varying ionospheric delays, the algorithm can still predict the ionospheric differences between epochs through the ionospheric prediction model to resolve false detections. Finally, the algorithm does not require complex search during cycle slip repair and can achieve efficient cycle slip detection and real-time repair with good accuracy and robustness.

The experimental results show that even during ionospherically active periods, the algorithm still has good detection performance for insensitive cycle slip combinations, especially small-cycle combinations. Also, during the cycle slip repair process, the algorithm can complete the repair without complex search.

Our future research will primarily focus on two crucial areas. Firstly, we intend to broaden our exploration to multiple satellite systems, such as GPS and Galileo. Simultaneously, we have observed that the positioning principles of BDS, GPS, and Galileo share a fundamental similarity. With the exception of BDS’s GEO satellites, the basic principles of other satellite systems align, providing a foundation for our approach to achieve consistent performance across different systems. This expansion aims to deepen our understanding of the versatility and effectiveness of our methodology across various satellite constellations. Secondly, we aim to assess how our approach performs in more disrupted ionospheric conditions, evaluating its resilience in intricate environments. These efforts are directed towards continually refining and advancing the cycle-slip detection technique, ensuring its adaptability across diverse satellite systems. This adaptability is crucial for the algorithm to consistently deliver stable and accurate performance in real-world applications, particularly in the face of challenging ionospheric scenarios.

## Data Availability

The authors confirm that the data supporting the findings of this study are available within the article and its supplementary materials. The supplementary materials include simulation data from the experiments and details on how to obtain them are provided in the file description document.
